# Sarcomatoid Squamous Cell Carcinoma and Its Mimics: A Meta-Analysis of Institutional Cases and Published Reports

**DOI:** 10.3390/cancers18091411

**Published:** 2026-04-29

**Authors:** Salin Kiratikanon, Yuqing Xiong, Jakob M. T. Moran, Mai P. Hoang

**Affiliations:** 1 Department of Pathology, Massachusetts General Hospital, Boston, MA 02114, USA; salin.k@cmu.ac.th (S.K.); yxiong13@mgh.harvard.edu (Y.X.); jmoran0@mgb.org (J.M.T.M.); 2 Division of Dermatology, Department of Internal Medicine, Faculty of Medicine, Chiang Mai University, Chiang Mai 50200, Thailand; 3 Harvard Medical School, Boston, MA 02115, USA

**Keywords:** sarcomatoid, squamous cell carcinoma, spindle, atypical fibroxanthoma, spindle cell melanoma, immunohistochemistry, systemic review, meta-analysis

## Abstract

**Background/Objective**: Sarcomatoid squamous cell carcinoma (sSCC) is a rare tumor that resembles atypical fibroxanthoma/pleomorphic dermal sarcoma (AFX/PDS) and spindle cell/dedifferentiated melanoma histologically. **Methods**: Immunohistochemistry was performed on 51 sSCCs from 46 patients, 26 AFX/PDS from 24 patients, and 15 spindle cell/dedifferentiated melanoma from 15 patients. Twenty-nine studies comprising 307 sSCCs, 636 AFX/PDS, and 168 spindle cell/dedifferentiated melanomas were included in the pooled analysis. **Results**: p63 showed the highest pooled sensitivity for sSCC (0.89), followed by keratin AE1/AE3 (0.87), keratin MNF116 (0.87), keratin 903 (0.85), p40 (0.82), and keratin 5/6 (0.72). Evidence regarding pooled diagnostic performance was limited for several markers. Among the best-supported markers for sSCC, p63 demonstrated a pooled OR of 42.36 (95% CI 13.95–128.61), sensitivity of 0.82, and specificity of 0.94; p40 showed a pooled OR of 50.27 (95% CI 13.91–181.70), sensitivity of 0.90, and specificity of 0.85; and keratin 5/6 had a pooled OR of 108.60 (95% CI 27.10–435.20), sensitivity of 0.94, and specificity of 0.93. For AFX/PDS, CD10 showed a pooled OR of 10.64 (95% CI 2.96–38.19), sensitivity of 0.73, and specificity of 0.80. For spindle cell/dedifferentiated melanoma, S100 showed a pooled OR of 161.23 (95% CI 24.55–1058.69), sensitivity of 0.95, and specificity of 0.94 (95% CI 0.85–0.97), while SOX10 yielded a pooled OR of 121.27 (95% CI 6.33–2323.34). **Conclusions**: A panel comprising p63 or p40, keratin 5/6, CD10, CD163 or CD68, and SOX10 or S100 may aid in distinguishing sSCC from AFX/PDS and spindle cell/dedifferentiated melanoma.

## Simple Summary

Sarcomatoid squamous cell carcinoma is a rare but aggressive cancer. In this study, we aimed to find laboratory tests to distinguish it from other histologically similar tumors. We found that a panel of immunostains, including p63 or p40, keratin 5/6, CD10, CD163 or CD68, and SOX10 or S100, can help to differentiate sarcomatoid squamous cell carcinoma from histopathologic mimics, such as atypical fibroxanthoma, pleomorphic dermal sarcoma, and spindle cell or dedifferentiated melanoma, leading to more accurate diagnosis in clinical practice.

## 1. Introduction

Squamous cell carcinoma (SCC) is the second most common cutaneous malignancy and commonly occurs on sun-exposed sites. Primary cutaneous sarcomatoid squamous cell carcinoma (sSCC), also referred to in the literature as spindle cell SCC and desmoplastic SCC, represents an uncommon variant characterized by spindle cell morphology [1,2]. Certain histologic subtypes of SCC—including sarcomatoid, desmoplastic, spindle cell, acantholytic, and adenosquamous variants—are associated with a higher risk of recurrence and metastasis [1]. Accurate distinction of sSCC from its histopathologic mimics, particularly atypical fibroxanthoma/pleomorphic dermal sarcoma (AFX/PDS) and certain subtypes of melanoma, is clinically important because immunotherapy is an established treatment option for advanced SCC and melanoma [3,4]. On the other hand, surgical excision is the main treatment for AFX/PDS.

sSCC has a spindled histomorphology and shows rare or absent keratinization. The histopathologic differential diagnosis of sSCC most commonly includes AFX/PDS and spindle cell/dedifferentiated melanoma. The histologic differential diagnosis can also include other entities such as leiomyosarcoma and angiosarcoma. Molecular analyses demonstrating ultraviolet signatures can be helpful in distinguishing SCC from mimics; however, access to molecular testing may be limited. In daily practice, the diagnosis is often established utilizing a panel of immunohistochemical (IHC) stains. Currently available immunostains have limitations. Although conventional SCC invariably expresses high-molecular-weight keratins such as keratin 903 (CK34βE12) and keratin 5/6, sSCC may exhibit only focal staining for these markers [5]. While p63 has been reported to be more sensitive than p40 in detecting sSCC, p40 is more specific than p63 in the distinction of sSCC from other spindle cell neoplasms [6,7].

To differentiate sSCC from other entities, other immunostains are needed. Spindle cell melanoma typically exhibits SOX10 and S100 positivity, while only focal or absent staining for MelanA, MiTF, and HMB-45 is seen. Dedifferentiated melanoma may lose both morphologic and immunophenotypic evidence of melanocytic differentiation, and a background of conventional melanoma is seen. Tumors with complete loss of melanocytic differentiation and no background conventional melanoma are termed undifferentiated melanomas; these entities are challenging to identify and often require molecular analysis [8]. Histiocytic markers such as CD68 and CD163 have been reported in the literature to stain AFX/PDS; however, these markers lack specificity, and the diagnosis of AFX/PDS is one of exclusion. Similarly, although CD10 is sensitive for AFX/PDS, it is not specific, as expression has been reported in 33% of desmoplastic melanomas and 50% of sSCC [9].

In this study, we present a cohort of institutional cases of sSCC and compare their immunoprofiles with those of AFX/PDS and spindle cell/dedifferentiated melanoma. We also reviewed previously published studies reporting on the IHC characteristics of these entities. By combining the results from our institutional cohort with data from the literature, we aim to derive a practical diagnostic IHC panel to aid in distinguishing sSCC from its histologic mimics. In addition, we explored prognostic factors associated with outcomes in sSCC.

## 2. Materials and Methods

### 2.1. Study Design and Data Collection

The study was approved by the institutional review board. We searched the pathology archives of Massachusetts General Hospital, Boston, for consecutive cases from 2000 to 2026. In this study, sSCC was defined as a poorly differentiated spindle or epithelioid cell tumor demonstrating evidence of epithelial differentiation, either through the presence of an associated well to moderately differentiated SCC component and/or definitive IHC expression of epithelial markers. The diagnoses of sSCC were established based on standard published histopathologic criteria and the results of IHC studies available at the time of diagnosis. Clinicopathological characteristics, immunoprofiles, and clinical outcomes were collected and reviewed. Patient demographics and clinical data—including age, sex, lesion location, date of diagnosis, date of recurrence, date of metastasis, and date of last follow-up—were obtained from medical records. Inclusion criteria consisted of patients aged ≥ 18 years with a diagnosis of sSCC at MGH made from January 2000 to February 2026. Cases lacking IHC results or with uncertain diagnoses were excluded. Comparator groups, including AFX/PDS and spindle cell and dedifferentiated melanoma, were also included. All spindle cell and dedifferentiated melanoma cases were confirmed by the identification of melanoma driver gene mutations involving *BRAF*, *NRAS*, *KIT*, *NF1*, or *TERT* through targeted DNA sequencing (Supplementary Figure S1).

### 2.2. Immunohistochemical Study

Immunohistochemical studies were performed on 5-micrometer-thick tissue sections using a Bond 3 automated immunostainer (Leica Microsystems, Bannockburn, IL, USA), with primary antibodies against p63 (4A4, predilute, Roche, Indianapolis, IN, USA), p40 (bc28, 1:100, BioCare, Temple, AZ, USA), keratin AE1/AE3 (ae1/ae3, predilute, BioSB, Santa Barbara, CA, USA), keratin MNF116 (mnf116, 1:400, Dako Agilent, Santa Clara, CA, USA), keratin CAM5.2 (cam5.2, predilute, Cell Marque, Rocklin, CA, USA), keratin 7 (RN7, predilute, Leica Microsystems), keratin 5/6 (d5/16 b4, 1:100, Dako), keratin 903 (CK34βE12, predilute, Leica Microsystems), pan-keratin (1:500, ThermoFisher Scientific, Carlsbad, CA, USA), GATA3 (undiluted, L50-823, Leica Microsystems), CD10 (56c6, predilute, Leica Microsystems), S100 (EP32, predilute, Leica Microsystems), SOX10 (SP267, predilute, Roche), MelanA (a103, predilute, Leica Microsystems), Mart1 (m2-7c10, 1:200, Cell Marque), MiTF (c5/d5, predilute, BioSB), CD68 (kp1, predilute, BioCare), CD163 (10d6, predilute, Leica Microsytems), and smooth muscle actin (alpha sm-1, predilute, Leica Microsystems). Appropriate positive and negative controls were run concurrently for the markers tested. Positivity is defined as staining greater than 10% of the tumor cells. The immunostains were evaluated by two pathologists, and discrepancies were resolved by consensus.

### 2.3. Literature Review

Studies were identified through a systematic search of PubMed or Embase databases from January 1990 to February 2026. The search aimed to identify studies reporting IHC results of sSCC, AFX/PDS, and spindle cell/dedifferentiated melanoma. The search strategy included combinations of the following search terms: (‘spindle cell squamous cell carcinoma’ OR ‘sarcomatoid squamous cell carcinoma’ OR ‘poorly differentiated squamous cell carcinoma’ AND ‘immunohistochemistry’ or ‘expression’ or ‘marker’); (‘atypical fibroxanthoma’ OR ‘pleomorphic dermal sarcoma’ AND ‘immunohistochemistry’ OR ‘expression’ OR ‘marker’); and (‘spindle cell melanoma’ OR ‘undifferentiated melanoma’ OR ‘dedifferentiated melanoma’ AND ‘immunohistochemistry’ OR ‘expression’ OR ‘marker’).

**Figure 1:**
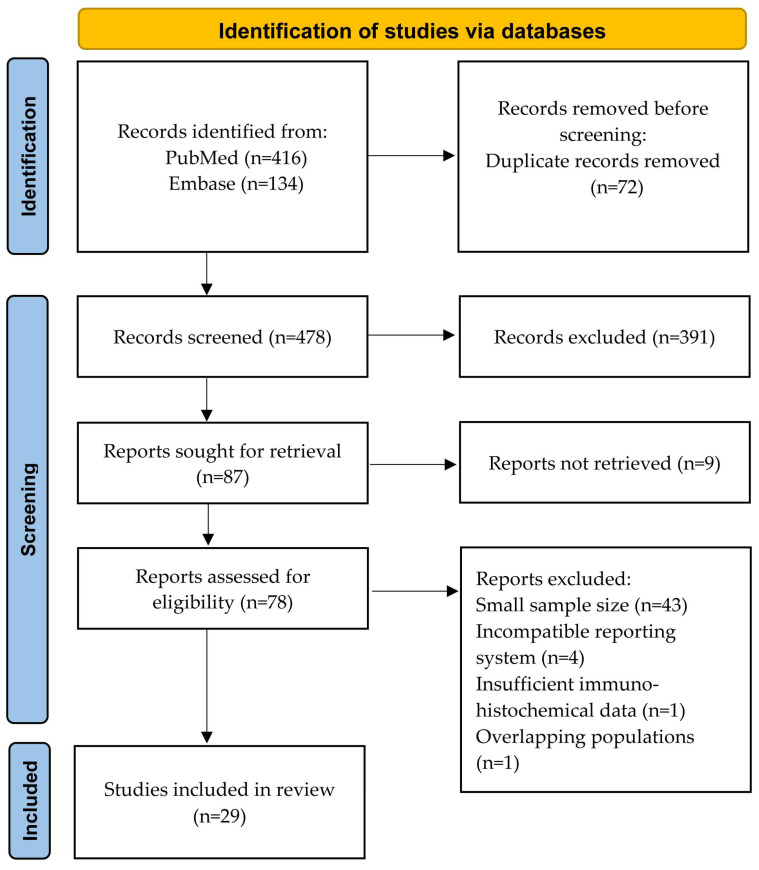
Preferred Reporting Items for Systematic Reviews and Meta-Analyses (PRISMA) flow diagram.

The titles and abstracts were screened to identify potentially relevant studies, followed by full-text review of selected manuscripts. Conference abstracts, non-English publications, and studies involving non-human subjects were not included. Studies with uncertain diagnoses and those with less than 10 included cases were excluded. To facilitate statistical analyses, published IHC results were recorded as positive or negative, based on the original classifications reported in each study. SK and MH independently performed the literature screening and manual review. The full search strategy and study selection process are summarized in Figure 1. A PRISMA checklist has been included as Supplementary Table S1 [10].

### 2.4. Statistical Analyses

Categorical variables were summarized as counts and percentages, while continuous variables were presented as mean ± standard deviation (SD) or median and interquartile range (IQR), as appropriate. Overall survival (OS) was defined as the time in months from the date of initial diagnosis to death from any cause. Survival curves were estimated using the Kaplan–Meier method and compared between subgroups using the log-rank test. Multivariate analyses were performed using Cox proportional hazards regression models by including all statistically significant covariates from univariate Cox models. The proportionality assumptions of the Cox models were tested. Analyses and plots were performed using the R statistical software, version 4.5.2 [11].

A meta-analysis of proportions was performed using the *metaprop* command with the Freeman-Tukey double arcsine transformation to estimate the pooled sensitivity of IHC stains. Pooled odds ratios (ORs) for the diagnostic performance of each IHC marker in differentiating tumors of interest from their mimics were calculated using the *meta esize* command with a DerSimonian and Laird random-effects model. Pooled diagnostic accuracy was assessed using a bivariate random-effects model implemented with the *metadta* command. Fixed-effects models were applied when the number of included studies was insufficient to support random-effects estimation. Heterogeneity was assessed using the I^2^ statistic, with values > 50% indicating substantial heterogeneity. For pooled diagnostic accuracy, studies with missing values in any of the true positive, false positive, false negative, or true negative values were excluded from the analyses. For analyses including two studies or fewer, heterogeneity was not assessed. Differences between subgroups were considered statistically significant at *p* < 0.05. The analyses were performed using Stata version 18.5 (StataCorp, College Station, TX, USA).

## 3. Results

### 3.1. Clinical and Histopathologic Summary of Institutional sSCC Cases

A total of 46 patients with 51 sSCC tumors were included in the study. The cohort comprised 35 males and 11 females. The patients’ ages ranged from 58 to 101 years (median, 81 years). Tumor locations were the head and neck (41/46, 89%), trunk (2/46, 4.3%), and extremities (3/46, 6.5%). Clinical follow-up was available for 44 patients, with a follow-up duration ranging from 1 to 212 months (median, 33 months). Fourteen patients (31.8%) experienced recurrence, and 16 patients (36.4%) died.

**Figure 2:**
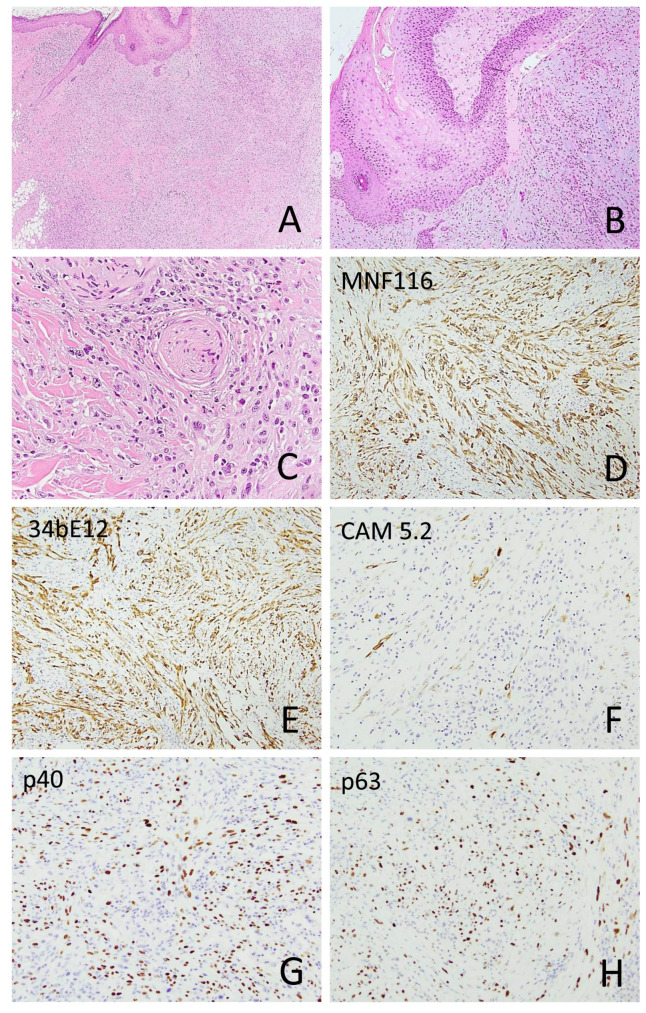
Sarcomatoid squamous cell carcinoma with overlying squamous cell carcinoma in situ ((**A**), 40×; (**B**), 100×) and perineural invasion ((**C**), 400×). The spindle tumor cells express keratin MNF116 ((**D**), 100×), keratin 34βE12 ((**E**), 100×), focally keratin CAM5.2 ((**F**), 200×), p40 ((**G**), 200×), and p63 ((**H**), 200×).

**Figure 3:**
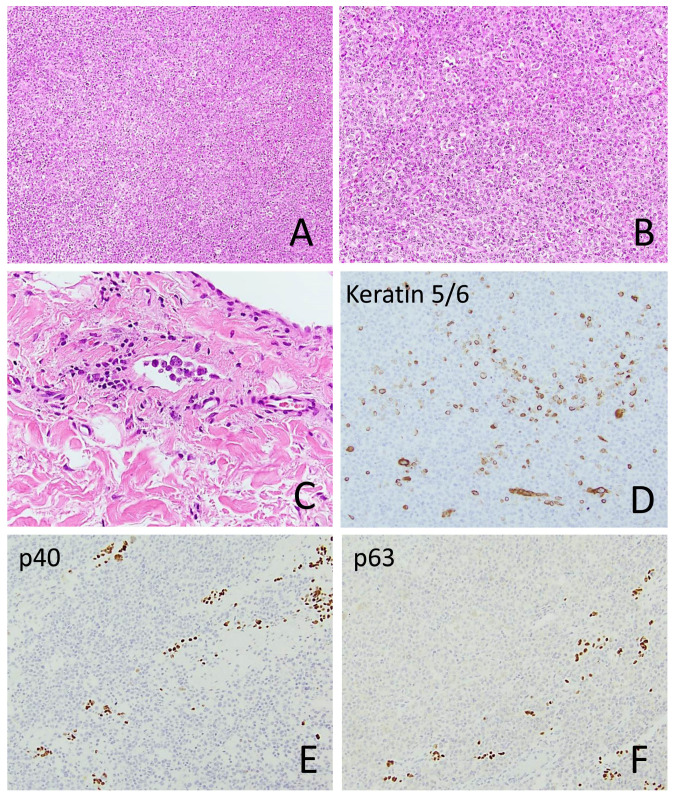
A sarcomatoid squamous cell carcinoma with an epithelioid morphology resembling melanoma ((**A**), 100×; (**B**), 200×) and lymphovascular invasion ((**C**), 400×). Only focal expressions of keratin 5 ((**D**), 200×), p40 ((**E**), 200×), and p63 ((**F**), 200×) are seen.

Among the sSCC tumors, six exhibited an in situ SCC component (Figure 2), and six had an associated moderately differentiated SCC component. Tumor size ranged from 0.3 cm to 10 cm (median, 1.4 cm). Perineural invasion and intravascular invasion were identified in 7/49 (14.3%) and 4/49 (8.2%) tumors, respectively (Figure 2 and Figure 3). The diameter of the involved nerve was <0.1 mm in six cases and >0.1 mm in one case. Multifocal perineural invasion was seen in two cases.

For comparison, 26 AFX/PDS tumors from 24 patients (17 males and 7 females) were included. The ages of these patients ranged from 61 to 98 years (median, 75 years). Tumor locations were the head and neck (25/26, 96.2%) and trunk (1/26, 3.9%). Fifteen cases of spindle cell melanoma or poorly differentiated/dedifferentiated melanoma with molecular confirmation were also included (Supplementary Figure S1). The ages of these patients ranged from 45 to 89 years (median, 68 years).

### 3.2. Survival Analyses of Sarcomatoid Squamous Cell Carcinoma Patients

**Figure 4:**
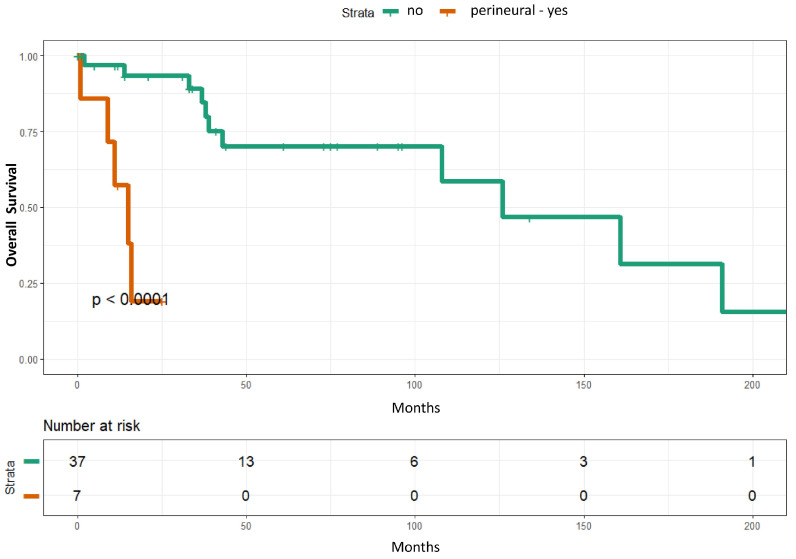
Kaplan–Meier curves of overall survival stratified by perineural invasion. Patients with perineural invasion exhibited significantly shorter overall survival compared with those without perineural invasion (*p* < 0.0001).

**Figure 5:**
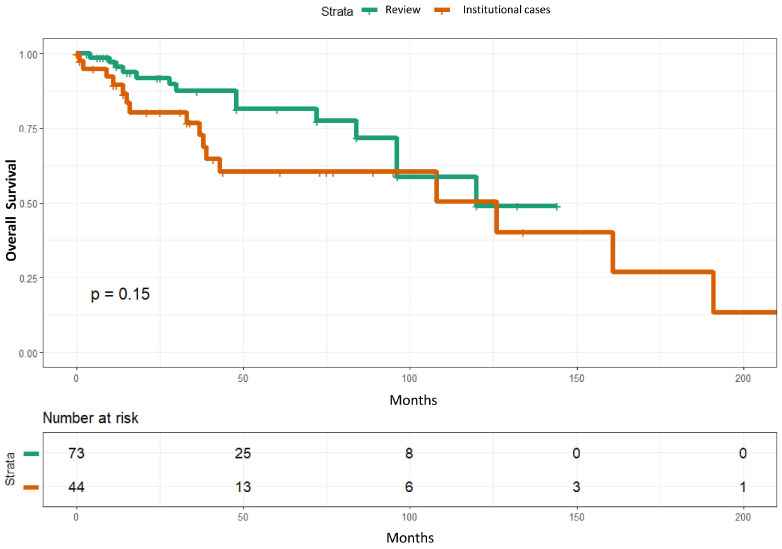
Kaplan–Meier curves showing comparable overall survival between the 44 institutional and 73 sarcomatoid squamous cell carcinoma cases reported in the literature [1,2,12].

In univariate analyses, perineural invasion (*p* = 0.0008) and lymphovascular invasion (*p* = 0.015) were significantly associated with overall survival. In multivariate analyses, only perineural invasion remained statistically significant (*p* = 0.003) (Table 1) (Figure 4). However, the event per variable is low, with perineural invasion identified in seven patients. Overall survival of the 44 institutional sSCC was not significantly different from that reported in 73 cases from the literature (*p* = 0.15) [1,2,12] (Figure 5).

### 3.3. Immunohistochemical Results of Institutional Cases

**Figure 6:**
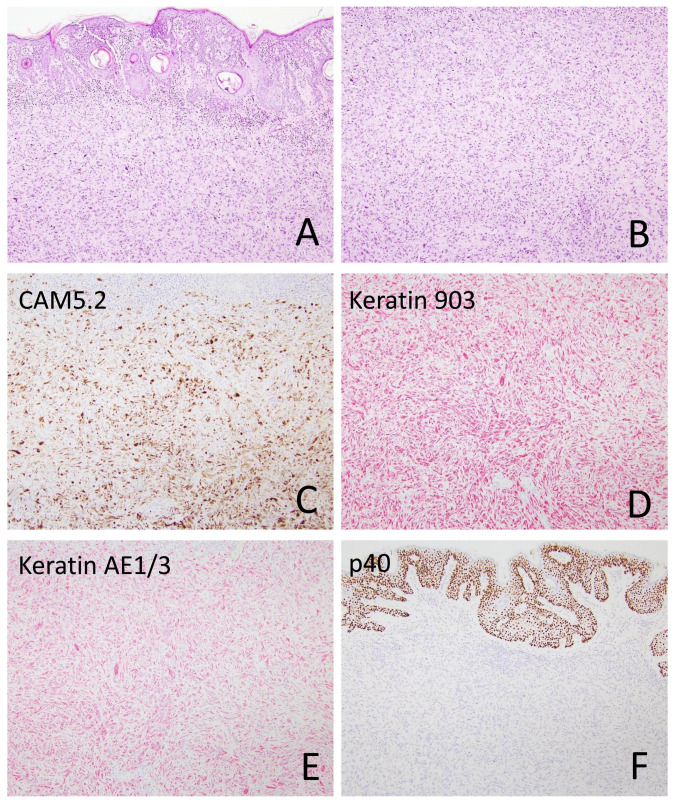
A sarcomatoid squamous cell carcinoma ((**A**), 40×; (**B**), 100×) strongly expresses keratin CAM5.2 ((**C**), 100×), keratin 903 (CK34βE12) ((**D**), 100×), and keratin AE1/3 ((**E**), 100×). While the overlying epidermis is positive for p40, the tumor cells are negative ((**F**), 100×).

Immunohistochemical stains were performed on 51 sSCC samples from 46 patients. Among epithelial markers, keratin 903 (CK34βE12) was positive in 15 of 18 cases (83.3%), followed by pan-keratin (16/20, 80%), keratin MNF116 (10/13, 76.9%), p63 (22/31, 71%), keratin 5/6 (23/33, 69.7%), keratin CAM5.2 (16/28, 57.1%), p40 (14/29, 48.3%), and keratin 7 (2/7, 28.6%). Keratin AE1/AE3 was positive in 2 of 2 cases, and GATA3 was positive in 3 of 3 cases. CD10 was positive in 15 of 16 cases (93.8%), and CD68 was positive in 12 of 18 cases (66.7%). Notably, none of the melanocytic markers—including S100, SOX10, and MelanA/Mart1—were positive in sSCC (Figure 2, Figure 3 and Figure 6; Table 2).

A total of 26 AFX/PDS samples from 24 patients were analyzed. CD10 was positive in all tested cases (18/18, 100%), followed by CD68 (7/9, 77.8%), SMA (4/11, 36.4%), CD163 (1/3, 33.3%), p63 (4/10, 40%), and S100 (1/15, 6.7%). GATA3 and MiTF were positive in two tested cases (Table 2).

In the spindle cell/dedifferentiated melanoma group, 15 samples were reviewed. Ten and five were spindle cell type and dedifferentiated melanoma, respectively. S100 was the most positive melanocytic marker (10/14, 71.4%), followed by SOX10 (8/15, 53.3%), PRAME (5/11, 45.5%), MiTF (4/10, 40%), and MelanA/Mart1 (2/14, 14.3%). CD10 was positive in 1 of 1 tested cases. Notably, keratin expression was observed in some spindle cell/dedifferentiated melanoma cases, including p63 (2/4, 50%), keratin 5/6 (1/4, 25%), pan-keratin (2/10, 20%), and keratin MNF116 (1/6, 6.7%) (Table 2).

### 3.4. Systematic Review and Pooled Immunohistochemical Analyses

Twenty-nine studies comprising 307 sSCC, 636 AFX/PDS, and 168 spindle cell/dedifferentiated melanoma cases were identified through the systematic search. After combining this data with cases from the institutional cohort, a total of 358 sSCC, 662 AFX/PDS, and 183 spindle cell/dedifferentiated melanoma cases were included in the pooled analysis. The PRISMA checklist is included as Supplementary Table S1.

**Figure 7:**
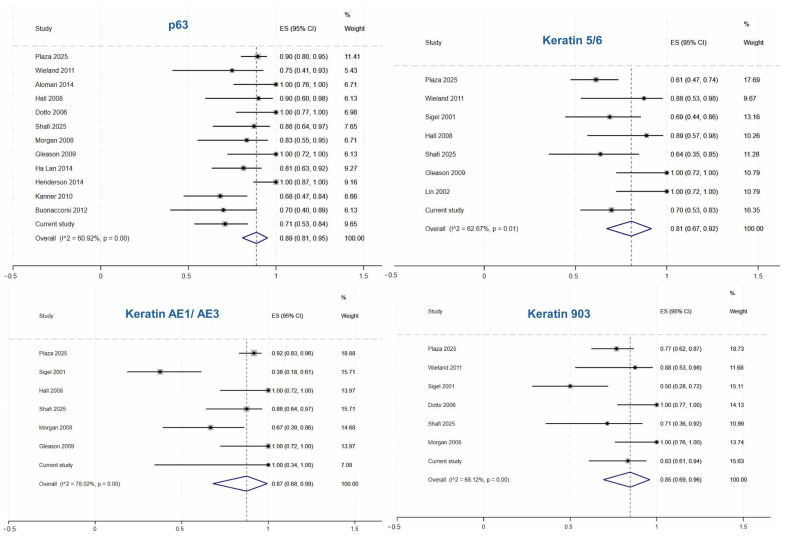
Forest plots of pooled sensitivity for p63, keratin 5/6, keratin AE1/AE3, and keratin 903 [1,6,7,13,14,15,16,17,18,19,20,21,22,26].

Table 3 summarizes the pooled sensitivity estimated by using a random-effects meta-analysis of proportions from combining the institutional cohort and the literature review. Among sSCC, the pooled sensitivity of p63 was the highest (0.89 [95% CI 0.81–0.95, I^2^ 60.92%]), followed by keratin AE1/AE3 (0.87 [95% CI 0.68–0.99, I^2^ 78.02%]) and keratin MNF116 (0.87 [95% CI 0.76–0.95, I^2^ 37.61]). For AFX/PDS, CD10 showed the highest pooled sensitivity (0.94 [95% CI 0.87–0.99, I^2^ 55.99%]), followed by CD68 (0.87 [95% CI 0.46–0.99, I^2^ 90.66%]), and CD163 (0.73 [95% CI 0.47–0.94]). In spindle cell/dedifferentiated melanoma, pooled sensitivity of PRAME was 0.81 (95% CI 0.60–0.96), followed by S100 (0.50 [95% CI 0.04–0.96, I^2^ 94.05%]). Figure 7 shows forest plots of the included studies and the pooled sensitivity estimate for selected stains in sSCC.

### 3.5. Diagnostic Performance

Table 4 summarizes the pooled diagnostic performance of immunostains in differentiating the tumors of interest from their mimics. In comparisons of sSCC versus AFX/PDS and spindle cell/dedifferentiated melanoma, multiple epithelial markers showed significant diagnostic utility. Marker p63 was the most extensively studied marker, demonstrating a pooled OR of 42.36 (95% CI 13.95–128.61), with a pooled sensitivity of 0.82 (95% CI 0.70–0.89) and specificity of 0.94 (95% CI 0.83–0.98). Heterogeneity was substantial for OR estimates (I^2^ = 55.66%) and moderate for sensitivity (I^2^ = 41.36%) and specificity (I^2^ = 35.82%). Keratin 5/6, derived from four studies, had a pooled OR of 108.60 (95% CI 27.10–435.20), sensitivity of 0.94 (95% CI 0.82–0.98), and specificity of 0.93 (95% CI 0.72–0.99). I^2^ was 0.0% for OR estimates, 0.50% for sensitivity (low heterogeneity) and 49.82% for specificity (moderate heterogeneity); however, interpretation was limited by the small number of included studies. Marker p40, analyzed based on four studies, showed a pooled OR of 50.27 (95% CI 13.91–181.70), sensitivity of 0.90 (95% CI 0.78–0.96), and specificity of 0.85 (95% CI 0.63–0.95). Heterogeneity for OR estimates was moderate (I^2^ = 30.48%), but low for sensitivity (I^2^ = 16.76%) and substantial for specificity (I^2^ = 65.16%).

Among other keratin markers, the available evidence was limited by the small number of included studies (two to three studies per marker). Keratin 903 showed a pooled OR of 178.10 (95% CI 25.07–1265.24), sensitivity of 1.00, and specificity of 0.90 (95% CI 0.72–0.97), with low heterogeneity. Keratin MNF116 demonstrated a pooled OR of 130.61 (95% CI 22.88–745.62), sensitivity of 0.97 (95% CI 0.73–1.00), and specificity of 0.91 (95% CI 0.76–0.97), with low heterogeneity. Keratin AE1/AE3 had a pooled OR of 362.21 (95% CI 34.76–3774.25), sensitivity of 0.96 (95% CI 0.84–0.99), and specificity of 0.93 (95% CI 0.72–0.99), without observed heterogeneity. Keratin CAM5.2 showed a pooled OR of 96.54 (95% CI 10.91–854.65), sensitivity of 1.00, and specificity of 0.75 (95% CI 0.62–0.85). Pan-keratin demonstrated a pooled OR of 39.64 (95% CI 9.42–166.88), sensitivity of 0.87 (95% CI 0.66–0.96), and specificity of 0.88 (95% CI 0.79–0.94). Keratin 7 did not show significant discriminatory value (OR 1.36, 95% CI 0.04–46.65; *p* = 0.863), and pooled sensitivity and specificity estimates were not available because of insufficient data.

In comparisons of AFX/PDS versus sSCC and spindle cell/dedifferentiated melanoma, CD10 demonstrated the strongest overall diagnostic utility (based on six studies), with a pooled OR of 10.64 (95% CI 2.96–38.19), pooled sensitivity of 0.73 (95% CI 0.62–0.82), and specificity of 0.80 (95% CI 0.55–0.93). Substantial heterogeneity was observed for OR estimates (I^2^ = 62.10%), whereas heterogeneity for sensitivity and specificity was moderate (I^2^ = 52.52% and 28.00% respectively). CD163, CD68 and SMA did not demonstrate significant discriminatory value. Diagnostic accuracy estimates were not available for CD68 and SMA because of insufficient data (Table 4).

To differentiate spindle cell/dedifferentiated melanoma from sSCC and AFX/PDS, only a few studies were included, resulting in limited interpretation of their diagnostic performances. S100 had a pooled OR of 161.23 (95% CI 24.55–1058.69), sensitivity of 0.95 (95% CI 0.73–0.99), and specificity of 0.94 (95% CI 0.85–0.97). I2 for OR estimates was 0.00% and heterogeneity for sensitivity and specificity was not available due to the limited number of included studies. From the two included studies, PRAME did not show significant discriminatory utility with a pooled OR of 6.10 (95% CI 0.65–56.92), sensitivity of 0.53 (95% CI 0.36–0.70), and specificity of 0.57 (95% CI 0.32–0.79). I^2^ for OR estimates was 0.00% and I^2^ for sensitivity and specificity were not available because of the very limited studies included. Results for SOX10, MelanA/MART1 and MiTF were based solely on data from the institutional cohort (Table 4).

Because of the limited number of included studies in some markers, several pooled estimates had wide confidence intervals, indicating limited precision and interpretation.

## 4. Discussion

Similar to our cohort, sSCC in the literature most commonly occurs in the head and neck region of older patients [1,2,12]. In a series of 210 patients with cutaneous SCC, grade unspecified, treated at MD Anderson Cancer Center, 16.2% (37/210) developed recurrence and 24.8% (52/210) died [37]. In our cohort, 31.8% (14/44) of sSCC patients experienced recurrence and 36.4% (16/44) died. The OS of the 44 institutional sSCC cases with available follow-up was comparable to that reported in 73 sSCC cases from the literature (Figure 3) [1,2,12]. Since poor histologic grade is a known predictor of adverse prognosis in cutaneous SCC [38], it is expected that sSCC, a poorly differentiated variant, is associated with worse outcomes than conventional SCC.

Perineural invasion in conventional cutaneous SCC has been shown to be associated with poor clinical outcomes [39]. In addition, perineural invasion has been reported as an independent adverse prognostic factor in desmoplastic SCC [40]. In conventional SCC, survival and recurrence rates are worse when the involved nerve has a diameter greater than 0.1 mm compared with smaller nerves [39,41]. In our institutional cohort, perineural invasion was associated with worse OS, and small-caliber peripheral nerves were involved in 86% (6/7) of these cases. Therefore, in contrast to observations in conventional SCC, our findings suggest that involvement of nerves of any caliber in sSCC may be associated with worse overall survival. However, the event per variable is less than ten due to the rarity of sSCC; therefore, additional study with a larger sample size is needed to confirm this observation.

**Figure 8:**
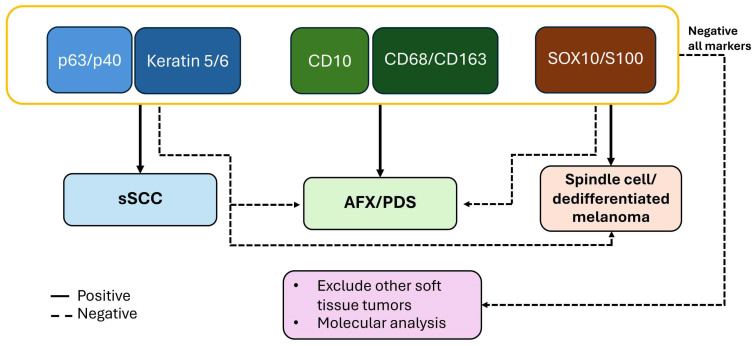
Proposed immunohistochemical panel.

Distinguishing sSCC from its histologic mimics, such as AFX/PDS and spindle cell/dedifferentiated melanoma, has important therapeutic implications, as immunotherapy is an approved treatment option for advanced SCC [3]. However, distinguishing sSCC, AFX/PDS, and spindle cell/dedifferentiated melanoma can be challenging given overlapping morphologic features. In routine diagnostic practice, the use of a panel of immunostains can improve diagnostic sensitivity and specificity. At the same time, the number of stains must be balanced with cost considerations, as a smaller, targeted panel is often more practical and cost-effective. Our analysis suggests that a panel consisting of nuclear epithelial markers (p63 or p40) and cytoplasmic epithelial markers (keratin 5/6) and CD10 with histiocytic markers (CD163 or CD68), and melanocytic markers (S100 or SOX10) is useful in distinguishing sSCC from AFX/PDS and spindle cell/dedifferentiated melanoma (Table 4; Figure 8). If the initial panel does not yield a definitive diagnosis, other entities such as leiomyosarcoma and angiosarcoma can be considered. Leiomyosarcoma is typically positive for smooth muscle markers such as SMA, desmin and h-caldesmon. Angiosarcoma is positive for vascular markers such as CD31 and ERG [5]. In some instances, molecular analyses are necessary for diagnosis.

In a series of 74 sSCC cases reported by Plaza et al., all tumors were positive for at least one epithelial marker, including keratin AE1/AE3 (91.6%), p63 (89%), keratin MNF116 (78%), p40 (77.5%), high-molecular-weight keratin (76.7%), keratin CAM5.2 (72%), and keratin 5/6 (61%) [1]. Keratin MNF116 detects both low- and high-molecular-weight keratins, including keratins 5, 6, 8, 17, and 19 [42]. Previous studies have suggested that keratin MNF116 may have greater sensitivity for detecting sSCC compared with keratin AE1/AE3, keratin 903 (CK34βE12), and keratin CAM5.2 [1,14]. In our study, the pooled sensitivities of keratin MNF116 were comparable to those of keratin AE1/AE3 and keratin 903 (CK34βE12), but their diagnostic performance remains limited due to the small number of included studies (Table 3 and Table 4).

The availability of p63 and p40 immunostains has improved the diagnosis of sSCC [19], since up to one-third of sSCC cases may lack expression of conventional keratin markers [22]. Consistent with our findings, although p40 is more specific, it is less sensitive than p63 for detecting sSCC (56% vs. 81%) [6,7]. While focal p63 expression has been reported in approximately 13% of AFX cases, p40 expression is observed in only about 3% of AFX cases (Table 3). In addition to basal cell carcinoma and adnexal neoplasms, p63 expression has also been reported in AFX, melanoma, and leiomyosarcoma [7,14,16]. Specifically, weak p63 positivity was observed in 2/10 (20%) AFX cases [16]. Other studies have reported p63 expression in AFX, for example, 3/23 (13%) [15], 5/31 (16%) [20], 1/15 (7%) [13], and 1/3 (33%) [18]. This phenomenon may be explained by the fact that p63 antibodies recognize all six p63 isoforms, whereas p40 recognizes only three [14].

Aberrant expression of mesenchymal markers can be observed in sSCC, with SMA expression reported in up to 40% of cases [17]. This may lead to misdiagnosis if an appropriate panel of IHC stains is not used. Conversely, keratin expression can also be detected in certain mesenchymal tumors, including epithelioid sarcoma, synovial sarcoma, epithelioid hemangioendothelioma, angiosarcoma, and epithelioid malignant peripheral nerve sheath tumor [5]. In addition, MiTF, a melanocytic marker, may be expressed in fibrohistiocytic tumors [43], including pleomorphic sarcoma [31]. Therefore, MiTF does not appear to be a specific marker.

From the pooled sensitivity analyses, a small subset of AFX/PDS cases expressed keratin markers, including p63, p40, keratin 5/6, and pan-keratin, whereas none expressed keratin AE1/AE3, keratin MNF116, keratin CAM5.2, or keratin 903 (CK34βE12). Similarly, a small number of spindle cell/dedifferentiated melanomas showed expression of keratin markers such as p63, p40, keratin MNF116, keratin CAM5.2, and keratin 5/6, while none expressed keratin AE1/AE3, keratin 7, or keratin 903 (CK34βE12) (Table 3).

The addition of melanocytic markers is necessary for identifying melanoma. S100 was expressed in 47.4% spindle cell/dedifferentiated melanoma cases. SOX10 is more specific but less sensitive and may therefore be used as a subsequent confirmatory marker. MiTF and PRAME are not entirely specific, as they may also be positive in AFX/PDS.

Markers such as CD10, CD68, and CD163 may support a diagnosis of AFX/PDS, but they are not entirely specific, and they may stain intratumoral histiocytes, not the tumor cells themselves. CD10 has been reported to be positive in melanoma [9,28] and sSCC [13]. In the literature, CD163 is expressed in approximately 79% of AFX cases but is absent in sSCC and is only rarely expressed in spindle cell or desmoplastic melanoma [24]. These findings are concordant with our cohort. Nevertheless, the diagnosis of AFX/PDS remains mainly one of exclusion.

Other spindle cell neoplasms should also be considered in the differential diagnosis, particularly angiosarcoma and leiomyosarcoma. Poorly differentiated angiosarcoma may lack well-formed vascular structures and typically arises in chronically sun-damaged skin. ERG is regarded as the most sensitive and specific marker for angiosarcoma, with reported sensitivity and specificity approaching 100%, and typically shows strong and diffuse expression [44,45]. In contrast, ERG expression has not been reported in poorly differentiated SCC, melanoma, or AFX [45,46,47]. Leiomyosarcoma, which rarely arises in chronically sun-damaged skin, typically shows immunoreactivity for SMA, desmin, vimentin, and h-caldesmon [18,48,49,50,51]. CD10 is usually negative [21]. Rare cases may demonstrate S100 positivity, and focal pan-keratin expression has been reported in approximately 45% of cases [50].

Based on our findings, we propose an initial IHC panel consisting of p63 or p40, keratin 5/6, CD10, and SOX10 or S100. P63 and p40 demonstrated both high sensitivity and specificity, with additional support from keratin 5/6. Although keratin AE1/AE3, keratin 903 (CK34βE12) and keratin MNF116 also showed favorable diagnostic performance, their diagnostic utility is limited by the small number of included studies. The addition of a melanocytic marker such as SOX10 or S100 can aid in identifying spindle cell/dedifferentiated melanoma. A stepwise IHC approach is illustrated in Figure 8. In diagnostically challenging cases, molecular testing plays an important role in establishing a definitive diagnosis.

### Strength and Limitations

A key strength of this study is the inclusion of a relatively substantial institutional cohort of 51 tumors, providing a meaningful dataset for evaluating the immunoprofiles of sSCC. Importantly, the study incorporated relevant comparator entities, including AFX/PDS and spindle cell/dedifferentiated melanoma, which represent the most common histologic mimickers of sSCC in routine diagnostic practice. In addition, the systematic review included a substantial number of previously reported cases from the literature, further strengthening the robustness and generalizability of the pooled analyses. Diagnostic performance metrics—including pooled OR estimates, sensitivity, and specificity—were assessed, allowing a comprehensive evaluation of the diagnostic utility of each marker. Based on these findings, we propose a practical IHC panel that may serve as an initial, comprehensive, and cost-effective guide in the diagnostic work-up of spindle cell cutaneous neoplasms.

Our study has several limitations. A major limitation is the heterogeneity among included studies, together with the limited number of studies available for the final diagnostic performance analyses. The heterogeneity may reflect differences in utilized antibody clones, staining protocols, and interpretation thresholds across institutions. Because these are rare tumors, we aimed to capture as much available evidence as possible through a systematic search and meta-analysis, although the overall data remained limited. Accordingly, the reported diagnostic performance estimates, particularly for markers supported by the sparse evidence, should be interpreted and applied cautiously in clinical practice and considered primarily exploratory findings that may complement routine diagnostic assessment. Furthermore, the proposed IHC panel should be considered only as an initial diagnostic guide. In challenging cases, additional epithelial markers may still be required to establish the diagnosis of sSCC confidently; other markers such as SMA, desmin, h-caldesmon, CD31 or ERG can also be considered to exclude other soft tissue tumors.

## 5. Conclusions

We present a cohort of sSCC to expand the current understanding of its clinical course and diagnostic challenges. We evaluated the diagnostic performance of commonly used IHC markers. Our results suggest that a focused panel including p63 or p40, keratin 5/6, CD10, CD163 or CD68, and S100 or SOX10 can aid in distinguishing these entities in routine practice. A stepwise and cost-conscious IHC approach may improve diagnostic accuracy, while molecular testing may provide additional support in diagnostically challenging cases.

## Figures and Tables

**Table 1:** Summary of univariate and multivariate Cox proportional hazards models for overall survival of institutional cases.

Variables	*N*	Univariate Analysis		Multivariate Analysis	
		HR (95% CI)	*p*-Value	HR (95% CI)	*p*-Value
Age	44	0.79 (0.29, 2.17)	0.65		
Sex	44	1.07 (0.33, 3.42)	0.91		
Site	44	0.56 (0.22, 1.47)	0.24		
Size	39	1.28 (0.81, 2.04)	0.29		
Perineural invasion	44	17.3 (3.29, 91.12)	0.0008 *	13.94 (2.43, 80.03)	0.003 *
Lymphovascular invasion	44	11.48 (1.6, 82.38)	0.015 *	3.57 (0.47, 27.34)	0.22

* *p*-value < 0.05, statistically significant, HR: hazard ratio, CI: confidence interval.

**Table 2:** Immunohistochemical staining results across tumor groups of institutional cases.

Stains	Sarcomatoid Squamous Cell Carcinoma		Atypical Fibroxanthoma/Pleomorphic Dermal Sarcoma		Spindle Cell/Dedifferentiated Melanoma	
	*n*/*N*	%	*n*/*N*	%	*n*/*N*	%
p63	22/31	71.0	4/10	40.0	2/4	50.0
p40	14/29	48.3	0/22	0.0	0/3	0.0
Keratin AE1/AE3	2/2	100.0	0/1	0.0	0/3	0.0
Keratin MNF116	10/13	76.9	0/17	0.0	1/6	16.7
Keratin CAM5.2	16/28	57.1	0/16	0.0	0/4	0.0
Keratin 7	2/7	28.6	-	-	0/1	0.0
Keratin 5/6	23/33	69.7	0/19	0.0	1/4	25.0
Keratin 903 (CK34βE12)	15/18	83.3	0/9	0.0	0/2	0.0
Pan-keratin	16/20	80.0	0/13	0.0	2/10	20.0
GATA3	3/3	100.0	2/2	100.0	-	-
CD10	15/16	93.8	18/18	100.0	1/1	100.0
CD68	12/18	66.7	7/9	77.8	1/3	33.3
CD163	1/3	33.3	1/3	33.3	0/1	0.0
SMA	3/12	25.0	5/12	41.7	1/9	11.1
S100	0/23	0.0	1/15	6.7	10/14	71.4
SOX10	0/27	0.0	0/26	0.0	8/15	53.3
MelanA/Mart1	0/19	0.0	0/6	0.0	2/14	14.3
MiTF	0/6	0.0	2/2	100.0	4/10	40.0
PRAME	-	-	0/1	0.0	5/11	45.5

*n*/*N* = number of positive cases/total tested.

**Table 3:** Pooled sensitivity of stains in sarcomatoid squamous cell carcinoma in current and previously published studies.

Stains	Sarcomatoid SCC		AFX		MM	
	Pooled Sensitivity (95% CI)	I^2^ (%)	Pooled Sensitivity (95% CI)	I^2^ (%)	Pooled Sensitivity (95% CI)	I^2^ (%)
p63	0.89 (0.81–0.95)	60.92	0.12 (0.03–0.24)	79.70	0.11 (0.00–0.30)	56.35
Keratin AE1/AE3	0.87 (0.68–0.99)	78.02	0.00 (0.00–0.00)	0.00	0.00 (0.00–0.25)	0.00
Keratin MNF116	0.87 (0.76–0.95)	37.61	0.00 (0.00–0.00)	0.00	0.17 (0.02–0.41)	NA
Keratin 903 (34βE12)	0.85 (0.69–0.96)	68.12	0.00 (0.00–0.00	0.00	0.00 (0.00–0.66)	NA
p40	0.82 (0.62–0.96)	85.06	0.01 (0.00–0.06)	7.25	0.15 (0.02–0.34)	NA
Keratin 5/6	0.81 (0.67–0.92)	62.67	0.01 (0.00–0.06)	0.00	0.06 (0.00–0.40)	0.00
Keratin CAM5.2	0.72 (0.59–0.83)	23.26	0.00 (0.00–0.00)	NA	0.04 (0.00–0.42)	0.00
Pankeratin	0.53 (0.22–0.84)	75.56	0.01 (0.00–0.06)	0.00	0.02 (0.00–0.23)	34.62
Keratin 7	0.26 (0.00–0.69)	87.72	NA	NA	0.00 (0.00–0.43)	NA
CD10	0.53 (0.25–0.81)	83.01	0.94 (0.87–0.99)	55.99	0.33 (0.00–0.86)	86.89
CD68	0.20 (0.00–0.72)	90.61	0.87 (0.46–0.99)	90.66	0.51 (0.18–0.84)	0.00
CD163	0.07 (0.00–0.41)	NA	0.73 (0.47–0.94)	NA	0.18 (0.00–0.64)	NA
SMA	0.50 (0.12–0.88)	92.35	0.53 (0.32–0.75)	79.38	0.06 (0.00–0.32)	NA
S100	0.00 (0.00–0.03)	0.00	0.05 (0.00–0.17)	91.85	0.50 (0.04–0.96)	94.05
SOX10	0.00 (0.00–0.12)	NA	0.00 (0.00–0.04)	NA	0.35 (0.00–0.95)	95.63
MiTF	0.00 (0.00–0.39)	NA	0.96 (0.77–1.00)	NA	0.40 (0.21–0.60)	44.94
MelanA/Mart1	0.00 (0.00–0.17)	NA	0.00 (0.00–0.00)	0.00	0.03 (0.00–0.15)	24.17
PRAME	-	NA	0.68 (0.39–0.92)	NA	0.81 (0.60–0.96)	NA
References	[1,6,7,13,14,15,16,17,18,19,20,21,22,23,24,25,26]		[6,7,8,9,13,14,15,16,19,20,21,24,25,27,28,29,30,31,32,33]		[7,8,9,20,21,23,24,25,32,34,35,36]	

AFX: atypical fibroxanthoma, CI: confidence interval, IHC: immunohistochemistry, NA: not available, OR: odds ratio, PDS: pleomorphic dermal sarcoma.

**Table 4:** Diagnostic performance of immunohistochemical stains in distinguishing the tumor of interest from its histologic mimics.

Comparison	Stain	OR (95% CI)	*p*-Value	I^2^ (%)	Sensitivity	I^2^ (%)	Specificity	I^2^ (%)	No. of Studies ^†^	References
sSCC versus AFX/PDS + spindle cell/ dedifferentiated melanoma	p63	42.36 (13.95–128.61)	<0.001 *	55.66	0.82 (0.70–0.89)	41.36	0.94 (0.83–0.98)	36.82	10	[6,7,13,14,15,16,19,20,21]
	Keratin 5/6	108.60 (27.10–435.20)	<0.001 *	0.00	0.94 (0.82–0.98)	0.50	0.93 (0.72–0.99)	49.82	4	[13,15,19]
	p40	50.27 (13.91–181.70)	<0.001 *	30.48	0.90 (0.78–0.96)	16.76	0.85 (0.63–0.95)	65.16	4	[6,7,14]
	Keratin 903 (34βE12)	178.10 (25.07–1265.24)	<0.001 *	0.00	1.00 (0.00–1.00)	0.00	0.90 (0.72–0.97)	8.71	3	[13,16]
	MNF116	130.61 (22.88–745.62)	<0.001 *	0.00	0.97 (0.73–1.00)	1.93	0.91 (0.76–0.97)	0.80	3	[14,16]
	Keratin AE1/AE3	362.21 (34.76–3774.25)	<0.001*	0.00	0.96 (0.84–0.99)	0.00	0.93 (0.72–0.99)	0.00	3 ^‡^	[15,19]
	Keratin CAM5.2	96.54 (10.91–854.65)	<0.001 *	0.00	1.00 (0.00–1.00)	NA	0.75 (0.62–0.85)	NA	2	[19]
	Pankeratin	39.64 (9.42–166.88)	<0.001 *	0.00	0.87 (0.66–0.96)	NA	0.88 (0.79–0.94)	NA	2	[21]
	Keratin 7	1.36 (0.04–46.65)	0.863	NA	NA	NA	NA	NA	NA	NA
AFX/PDS versus sSCC + spindle cell/ dedifferentiated melanoma	CD10	10.64 (2.96–38.19)	<0.001 *	62.10	0.73 (0.62–0.82)	52.52	0.80 (0.55–0.93)	28.00	6	[9,13,15,20,21]
	CD163	7.53 (0.72–78.82)	0.092	38.44	0.80 (0.53–0.93)	NA	0.72 (0.48–0.88)	NA	2	[24]
	CD68	2.15 (0.36–13.05)	0.404	NA	NA	NA	NA	NA	NA	NA
	SMA	3.04 (0.62–14.77)	0.169	NA	NA	NA	NA	NA	NA	NA
Spindle cell/ dedifferentiated melanoma versus sSCC + AFX/PDS	S100	161.23 (24.55–1058.69)	<0.001 *	0.00	0.95 (0.73–0.99)	NA	0.94 (0.85–0.97)	NA	2	[25]
	SOX10	121.27 (6.33–2323.34)	0.001 *	NA	NA	NA	NA	NA	NA	NA
	MelanA/ Mart1	10.20 (0.46–228.89)	0.143	NA	NA	NA	NA	NA	NA	NA
	MiTF	2.00 (0.26–15.38)	0.505	NA	NA	NA	NA	NA	NA	NA
	PRAME	6.10 (0.65–56.92)	0.113	0.00	0.53 (0.36–0.70)	NA	0.57 (0.32–0.79)	NA	2	[8]

* Statistically significant; ^†^ include data from the current study; ^‡^ all included studies reported 100% sensitivity and specificity; therefore, continuity correction was applied for meta-analysis. AFX: atypical fibroxanthoma, CI: confidence interval, IHC: immunohistochemistry, OR: odds ratio, PDS: pleomorphic dermal sarcoma, sSCC: sarcomatoid squamous cell carcinoma.

## Data Availability

All data supporting the findings of this study are available within the paper and Supplementary Information.

## Supplementary Materials

The following supporting information can be downloaded at: https://www.mdpi.com/article/10.3390/cancers18091411/s1, Figure S1: Summary of molecular alterations of 15 spindle cell/dedifferentiated melanomas. Results were obtained from targeted DNA sequencing, which utilized Anchored Multiplex PCR (polymerase chain reaction) (AMP) for single-nucleotide variant (SNV), insertion/deletion (indel), and copy number detection in genomic DNA using the ArcherDx platform and Illumina NextSeq next generation [PMID: 25384085]. The SNV/indel and CNV gene targets covered by this test are as follows (coding sequence): ABL1, ACVR1, AKT1, AKT2, AKT3, ALK, APC, AR, ARID1A, ARID1B, ARID2, ATM, ATR, ATRX, AURKA, B2M, BAP1, BARD1, BCOR, BLM, BMPR1A, BRAF, BRCA1, BRCA2, BRIP1, CCND1, CCND2, CCND3, CCNE1, CDH1, CDK12, CDK4, CDK6, CDKN2A, CDKN2B, CHD1, CHEK1, CHEK2, CIC, CSF1R, CTNNB1, DAXX, DDR2, DDX3X, DICER1, EGFR, EIF1AX, EP300, EPCAM, ERBB2, ERBB3, ERBB4, ERCC1, ERCC2, ESR1, EZH2, FANCA, FANCI, FANCL, FBXW7, FGF19, FGFR1, FGFR2, FGFR3, FGFR4, FH, FLCN, FLT1, FLT3, FLT4, FOXA1, FOXL2, FUBP1, GNA11, GNAQ, GNAS, H3F3A, H3F3B, HIST1H3B, HIST1H3C, HNF1A, HRAS, IDH1, IDH2, JAK1, JAK2, JAK3, KDM6A, KDR, KEAP1, KIT, KLF4, KMT2C, KMT2D (MLL2), KRAS, LZTR1, MAP2K1 (MEK1), MAP2K2 (MEK2), MAP3K1, MDM2, MDM4, MED12, MEN1, MET, MLH1, MPL, MRE11A, MSH2, MSH3, MSH6, MTAP, MTOR, MUC16, MUTYH, MYC, MYCN, MYOD1, NBN, NF1, NF2, NKX2-1, NOTCH1, NOTCH2, NOTCH3, NOTCH4, NPM1, NRAS, NTRK1, NTRK2, NTRK3, PALB2, PBRM1, PDGFRA, PDGFRB, PIK3CA, PIK3CB, PIK3R1, PLCB4, PMS2, POLD1, POLE, PPP2R1A, PPP2R2A, PRKD1, PTCH1, PTEN, PTPN11, RAD50, RAD51, RAD51B, RAD51C, RAD51D, RAD54L, RAF1, RB1, RET, RHOA, RICTOR, RNF43, ROS1, SDHA, SDHB, SDHC, SDHD, SETD2, SF3B1, SMAD2, SMAD4, SMARCA4, SMARCB1, SMO, SRC, SRSF2, STAG2, STK11, SUFU, TERT, TGFBR2, TP53, TP63, TRAF7, TSC1, TSC2, TSHR, U2AF1, VHL, XRCC2, and XRCC3. Table S1: PRISMA checklist [10].

## Abbreviations

The following abbreviations are used in this manuscript:

AFX	Atypical fibroxanthoma
IHC	Immunohistochemistry
MiTF	Microphthalmia-associated transcription factor
sSCC	Sarcomatoid squamous cell carcinoma
PDS	Pleomorphic dermal sarcoma
